# Impact of m6A demethylase (ALKBH5, FTO) genetic polymorphism and expression levels on the development of pulmonary tuberculosis

**DOI:** 10.3389/fcimb.2022.1074380

**Published:** 2022-12-22

**Authors:** Tian-Ping Zhang, Rui Li, Li-Jun Wang, Hong-Miao Li

**Affiliations:** ^1^ Department of Rheumatology and Immunology, The First Affiliated Hospital of USTC, Division of Life Sciences and Medicine, University of Science and Technology of China, Hefei, Anhui, China; ^2^ Department of Nosocomial Infection Management, The First Affiliated Hospital of Anhui Medical University, Hefei, China; ^3^ Department of Infectious Diseases, The First Affiliated Hospital of Anhui Medical University, Hefei, Anhui, China; ^4^ Department of Epidemiology and Biostatistics, School of Public Health, Anhui Medical University, Hefei, Anhui, China

**Keywords:** m6A demethylase, ALKBH5, FTO, pulmonary tuberculosis, single nucleotide polymorphisms

## Abstract

**Objective:**

The m6A methylation was involved in the pathogenesis of pulmonary tuberculosis (PTB), and our study aimed to reveal the potential association of m6A demethylase (ALKBH5, FTO) genes variation, expression levels and PTB.

**Methods:**

Eight SNPs (*ALKBH5* gene rs8400, rs9913266, rs12936694, rs4925144 and *FTO* gene rs6499640, rs8047395, rs1121980, rs9939609) were selected for genotyping by SNPscan technique in 449 PTB patients and 463 healthy controls.

**Results:**

The mRNA expression levels of ALKBH5, FTO were detected by qRT-PCR. There were no significant differences in genotype, allele distributions of all SNPs between PTB patients and healthy controls. Haplotype analysis demonstrated that the frequency of *FTO* gene GAAA haplotype was significantly reduced in PTB patients when compared to controls. *ALKBH5* rs8400 AA genotype, A allele frequencies were associated with the decreased risk of sputum smear-positive, while AA genotype frequency was related to the increased risk of hypoproteinemia in PTB patients. In addition, rs9913266 variant was linked to the occurrence of drug-induced liver injury, sputum smear-positive, and rs4925144 variant was associated with leukopenia among PTB patients. In *FTO* gene, rs8047395 GG genotype and G allele frequencies were significantly higher in the PTB patients with drug resistance than that in the PTB patients without drug resistance. The ALKBH5, FTO expression levels were significantly decreased in PTB patients in comparison to controls. Moreover, ALKBH5 level was increased in PTB patients with drug resistance, and FTO level was decreased in PTB patients with sputum smear-positive.

**Conclusion:**

*FTO* gene polymorphisms might be associated with PTB susceptibility, and ALKBH5, FTO levels were decreased in PTB patients, suggesting that these m6A demethylase played important roles in PTB.

## Introduction

As a common chronic infectious disease caused by *Mycobacterium tuberculosis* (MTB), TB is a major public health problem in developing countries and a great threat to human health (Glaziou et al., 2018). According to the latest World Health Organization report, there is an estimated 9.9 million new cases of TB worldwide in 2021, and China is one of the countries with high TB burden (World Health Organization, 2021). Studies had shown that although a quarter of the world’s population were infected with MTB, while only 10% of those infected population eventually develop active TB (Moonan et al., 2020; Luo et al., 2022). This suggested that a large number of the infected population might be naturally resistant to MTB infection, and host genetic susceptibility played non-negligible roles in TB development. In recent years, many studies had provided important clues to explore the roles of host genetic polymorphism in susceptibility to pulmonary TB (PTB) (Zhang et al., 2021; Chen et al., 2021). However, these results could only explain part of the heritability of PTB, and further exploration of the relationship between gene variations and PTB susceptibility would help to comprehensively understand the pathogenesis of MTB infection.

N6-methyladenosine (m6A) was the most common and enriched mRNA post-transcriptional modification, and widely distributed in prokaryotes and eukaryotes (Roundtree et al., 2017; Chen and Wong, 2020). M6A modification related enzymes mainly composed of m6A methyltransferase (METTL3, METTL14), m6A demethylase (FTO, ALKBH5) and m6A-binding protein (IGF2BP1, YTHDF1) (Hua et al., 2020). The m6A modification played important roles in a variety of biological processes, including mRNA translation, cell differentiation, immune response (Zhao et al., 2017) and dysregulated m6A modification was closely linked to various diseases, including autoimmune diseases, cancer (Han et al., 2019; Wang et al., 2021). The genetic variation in m6A-related genes might affect m6A modification level through altering the RNA sequence of target sites or key flanking nucleotides, and was considered to be a novel, important perspective for studying the disease susceptibility (Hua et al., 2020; Lin et al., 2020; Ruan et al., 2021).

FTO and ALKBH5 were the main m6A demethylases, which could reverse RNA methylation by oxidizing the N-methyl group at m6A site to a hydroxymethyl group, and were involved in the development of immune diseases and cancer (Wu et al., 2021; Deng et al., 2022). In addition, several typical single nucleotide polymorphisms (SNP) in *FTO* (rs9939609, rs1121980), *ALKBH5* (rs9913266, rs12936694) genes had been reported to affect the risk of gastric cancer, type 2 diabetes, autoimmune thyroid disease (Song et al., 2021; Binh et al., 2022; Li et al., 2022). PTB was also considered an immune-related infectious disease because the susceptibility of host to MTB was affected by the immune function (Feng et al., 2014; Liu et al., 2017). We speculated that *FTO*, *ALKBH5* gene variations might be involved in the immune response to MTB, and a few studies also suggested a key role of *FTO* gene variation in the risk of PTB (Feng et al., 2014; Naderi et al., 2017). It was possible that *ALKBH5* gene SNP also had a similar role in PTB, but relevant study was limited. Therefore, we performed this case–control study to assessed the association of *ALKBH5*, *FTO* genes polymorphisms, as well as their levels, with PTB susceptibility in a Chinese population.

## Materials and methods

### Study subjects

The PTB patients were recruited from the Department of Tuberculosis at Anhui Chest Hospital, and the healthy volunteers were enrolled from health examine center in the same geographical region as control group. The patients with PTB were diagnosed by experienced clinical specialists based on the following criteria: suspicious clinical symptoms, chest radiographs, sputum and/or bronchoalveolar lavage fluid MTB cultures, acid-fast bacilli (AFB) microscopy, and anti-TB treatment effectiveness. The exclusion criteria of PTB patients included HIV positive, hepatitis, malignancy and immunodeficiency. The control group needed to meet the following conditions: no history of TB, malignant tumor, HIV and other infectious diseases, negative sputum smear and culture, normal chest radiographs.

Our study was approved by the Medical Ethics Committee of Anhui Medical University (20200250). After obtaining the written informed consent of all participants, the peripheral blood samples, clinical data and demographic data of each participant were collected with the help of professional physicians. The required clinical data of this study included drug resistance, pulmonary infection, drug-induced liver injury (DILI), fever, leukopenia, sputum smear, total bilirubin (TBIL), alanine aminotransferase (ALT), erythrocyte sedimentation rate (ESR), aspartate aminotransferase (AST).

### DNA extraction and genotyping

In this study, we mainly adopted the pairwise option of HaploView 4.0 soft-ware (Cambridge, MA, USA) to screen Tag SNPs of *ALKBH5* and *FTO* genes by using the genotype data on *ALKBH5* and *FTO* genes of Han Chinese people in Beijing in two databases (Ensembl Genome Browser 85 and CHBS_1000g). Then, a comprehensive review of current studies on the association between *ALKBH5* and *FTO* genes polymorphisms and human diseases was conducted to search the potentially functional Tag SNPs, which were related to human diseases susceptibility. In addition, the candidate Tag SNPs were also identified according to the following criteria: (a) located at the two ends of these genes (ie, 5’ UTR, 3 ‘UTR, 5 ‘near gene, 3’ near gene); (b) the minor allele frequency (MAF) reported in 1000 Genomes ≥ 5% in CHB; (c) threshold *r^2^* > 0.8. As a result, we selected four Tag SNPs (rs8400, rs9913266, rs12936694, rs4925144) in *ALKBH5* gene and four Tag SNPs (rs6499640, rs8047395, rs1121980, rs9939609) in *FTO* gene, respectively.

Genomic DNA was extracted from peripheral blood samples using the Flexxi gene-DNA Kit (Qiagen, Valencia, CA) and frozen at −20°C until genotyping. With the technical support of the Center for Genetic & Genomic Anal-ysis, Genesky Biotechnologies (Inc., Shanghai), this study adopted SNPscan Kit for the genotyping of all SNPs and the specific steps could be found in the previous study (Peng et al., 2017).

### Quantitative real-time reverse transcription polymerase chain reaction

We isolated peripheral blood mononuclear cell (PBMC) from 3 ml anticoagulant peripheral blood, and then extracted total RNA from PBMC using TRIzol Reagent (Invitrogen, Carlsbad, CA, USA). The NanoDrop 2000 spectrophotometer (Thermo Scientific, USA) was used to determine the concentration of total RNA. Finally, the PrimeScriptTM RT Reagent Kit (Takara Bio Inc, Japan) was used for reverse transcription of total RNA to cDNA.

The ALKBH5, FTO mRNA levels in PBMC from PTB patients and controls were detected using qRT-PCR by SYBR Green (SYBR Premix Delivered Taq II, Legume Biotechnology Company, Japan), which was conducted in the QuantStudio 12 K Flex real-time PCR system (Applied Biosystems, Nurturing City,CA). The primer sequences of these two genes were listed as follow: ALKBH5, F: 5’-TCATCAACGACTACCAGCC-3’, R: 5’-GAAGGACACGGACACGAT 3’, FTO, F: 5’-TGGGTTCATCCTACAACGG-3’, R: 5’-CCTCTTCAGGGCCTTCAC-3’. The cycle conditions of qRT-PCR reactions were set as follow: 95°C for 1 min, followed by 42 cycles at 95°C for 10 sec, 60°C for 30 sec and 72°C for 1 min. In this study, we calculated the relative expression level of ALKBH5, FTO by comparison with the internal control gene β-actin in the same sample and used 2^-△△Ct^ method to express their levels.

### Statistical analysis

All statistical analyses were completed in SPSS 23.0 (SPSS Inc, IL, USA). Chi-square (*χ^2^*) test was used to evaluate whether each SNP satisfied the Hardy-Weinberg equilibrium (HWE) test in control group. Logistic regression analysis was performed to assess the relationship between all SNPs and PTB risk, and odds ratios (OR) and 95% confidence intervals (CI) were calculated to determine the statistical association. We also investigated the association of all SNPs on PTB susceptibility under two genetic models (dominant and recessive), and conducted haplotype analysis using SHEsis software (Li et al., 2009). The ALKBH5, FTO expression levels were expressed as median and quartile intervals, and the differences of ALKBH5, FTO expression levels between two groups, three groups were compared by Mann-Whitney *U* test, Kruskal-Wallis *H* test, respectively. The correlations between these m6A demethylase levels and experimental indexes of PTB patients were analyzed by Spearman rank correlation coefficient test. All tests for statistical significance used a two-sided *P* < 0.05.

## Results

### Association between *ALKBH5*, *FTO* genes polymorphisms and susceptibility to PTB

In this study, a total of 449 PTB patients (188 females and 261 males) and 463 healthy controls (201 females and 262 males) were selected for genotyping. The average ages of PTB patients and healthy controls were 45.78 ± 17.71 years and 43.27 ± 13.83 years, respectively. The allele, genotype frequencies of *ALKBH5* gene rs8400, rs9913266, rs12936694, rs4925144 variants, *FTO* gene rs6499640, rs8047395, rs1121980, rs9939609 were presented in **Table S1**, and the genotype distributions of above SNPs in healthy control were satisfied with HWE. The statistical power of all SNPs was in the range of 85% to 95%.

In *ALKBH5* gene, the allele frequencies of rs8400, rs9913266, rs12936694, rs4925144 variants were not associated with PTB susceptibility. In addition, there were no significant differences in genotype distributions of these SNPs between PTB patients and healthy controls (all *P* >0.05). When comparing the differences of genotype and allele frequencies of *FTO* gene rs6499640, rs8047395, rs1121980 and rs9939609 variants between PTB patients and healthy controls, we still did not find any statistically significant association. Similarly, none of these SNPs was related to PTB susceptibility under dominant, recessive models.

The relationships between *ALKBH5*, *FTO* genes variations and multiple clinical manifestations of PTB patients, including fever, drug resistance, DILI, pulmonary infection, hypoproteinemia, leukopenia, sputum smear-positive, were analyzed and listed in **Table S2**. We found that A allele, AA genotype frequencies of *ALKBH5* rs8400 were associated with the decreased risk of sputum smear-positive (*P* = 0.015, *P* = 0.019, respectively), and AA genotype frequency of rs8400 was related to the increased risk of hypoproteinemia in PTB patients (*P* = 0.023) (**Table 1**). In addition, *ALKBH5* rs9913266 A allele demonstrated a significant relationship with the increased occurrence of DILI (*P* = 0.046), as well as the decreased occurrence of sputum smear-positive (*P* = 0.049), and rs4925144 TT genotype seemed to be a protective factor for leukopenia in PTB patients (*P* = 0.019). In *FTO* gene, rs8047395 GG genotype and G allele frequencies were significantly higher in PTB patients with drug resistance than that in PTB patients without this clinical manifestation (*P* = 0.022, *P* = 0.008, respectively) (**Table 1**). There was no significant association between *ALKBH5* gene rs12936694, *FTO* gene rs6499640, rs1121980, rs9939609 variants and the clinical manifestations among PTB patients.

**Table 1:** The positive findings of association between *ALKBH5*, *FTO* gene polymorphism and some clinical manifestations in PTB patients.

SNP	Allele	Clinical features	Group	Genotype n (%)			*P* value	Allele n (%)		*P* value
	(M/m)			MM	Mm	mm		M	m	
ALKBH5										
rs8400	G/A	hypoproteinemia	+	12 (31.58)	14 (36.84)	12 (31.58)	**0.023**	38 (50.00)	38 (50.00)	0.156
			–	131 (31.87)	218 (53.04)	62 (15.09)		480 (58.39)	342 (41.61)	
		sputum smear-positive	+	99 (34.62)	152 (53.15)	35 (12.24)	**0.015**	350 (61.19)	222 (38.81)	**0.019**
			–	35 (28.23)	60 (48.39)	29 (23.39)		130 (52.42)	118 (47.58)	
rs9913266	G/A	DILI	+	26 (39.39)	32 (48.48)	8 (12.12)	0.120	84 (63.64)	48 (36.36)	**0.046**
			–	203 (53.00)	147 (38.38)	33 (8.62)		553 (72.19)	213 (27.81)	
		sputum smear-positive	+	71 (57.26)	44 (35.48)	9 (7.26)	0.141	186 (75.00)	62 (25.00)	**0.049**
			–	134 (46.85)	122 (42.66)	30 (10.49)		390 (68.18)	182 (31.82)	
rs4925144	C/T	leukopenia	+	7 (23.33)	21 (70.00)	2 (6.67)	**0.019**	35 (58.33)	25 (41.67)	0.252
			–	184 (43.91)	182 (43.44)	53 (12.65)		550 (65.63)	288 (34.37)	
FTO										
rs8047395	A/G	drug resistance	+	16 (21.62)	42 (56.76)	16 (21.62)	**0.022**	74 (50.00)	74 (50.00)	**0.008**
			–	140 (37.33)	183 (48.8)	52 (13.87)		463 (61.73)	287 (38.27)	

+/-: with/without Bold value means P < 0.05.

### Haplotype analysis

**Table 2** showed the frequency distributions of four main haplotypes (AGAT, AGGC, GAAC, GGAC) in *ALKBH5* gene and seven main haplotypes (AAAA, AAGT, AGGT, GAAA, GAAT, GAGT, GGGT) in *FTO* gene, which were identified by SHEsis software. The results suggested that the frequency of *FTO* gene GAAA haplotype was significantly reduced in PTB patients when compared to healthy controls (*P* = 0.026). While other haplotype frequencies were not associated with PTB susceptibility.

**Table 2:** Haplotype analysis of *ALKBH5*, *FTO* genes in PTB patients and controls.

Haplotype	PTB [n (%)]	Controls [n (%)]	*P* value	*OR* (95% CI)
*ALKBH5* rs8400-rs9913266-rs12936694-rs4925144				
AGAT	308.97 (34.4)	304.96 (32.9)	0.490	1.071 (0.881,1.301)
AGGC	64.99 (7.2)	79.99 (8.6)	0.273	0.826 (0.587,1.162)
GAAC	260.99 (29.1)	279.98 (30.2)	0.597	0.947 (0.774,1.159)
GGAC	251.97 (28.1)	250.99 (27.1)	0.634	1.051 (0.856,1.292)
*FTO* rs6499640- rs8047395-rs1121980-rs9939609				
AAAA	32.83 (3.7)	29.01 (3.1)	0.542	1.171 (0.704,1.947)
AAGT	68.72 (7.7)	65.97 (7.1)	0.674	1.078 (0.759,1.532)
AGGT	37.37 (4.2)	52.70 (5.7)	0.130	0.718 (0.467,1.104)
GAAA	42.74 (4.8)	66.91 (7.2)	**0.026**	0.640 (0.431,0.951)
GAAT	46.88 (5.2)	39.74 (4.3)	0.356	1.226 (0.795,1.890)
GAGT	345.76 (38.5)	374.02 (40.4)	0.391	0.921 (0.763,1.112)
GGGT	318.15 (35.4)	290.27 (31.3)	0.068	1.199 (0.987,1.458)

frequency < 0.03 in both controls & PTB patients has been dropped.

Bold value means P < 0.05.

### Expression levels of ALKBH5, FTO in PTB patients and healthy controls

**Figure 1:**
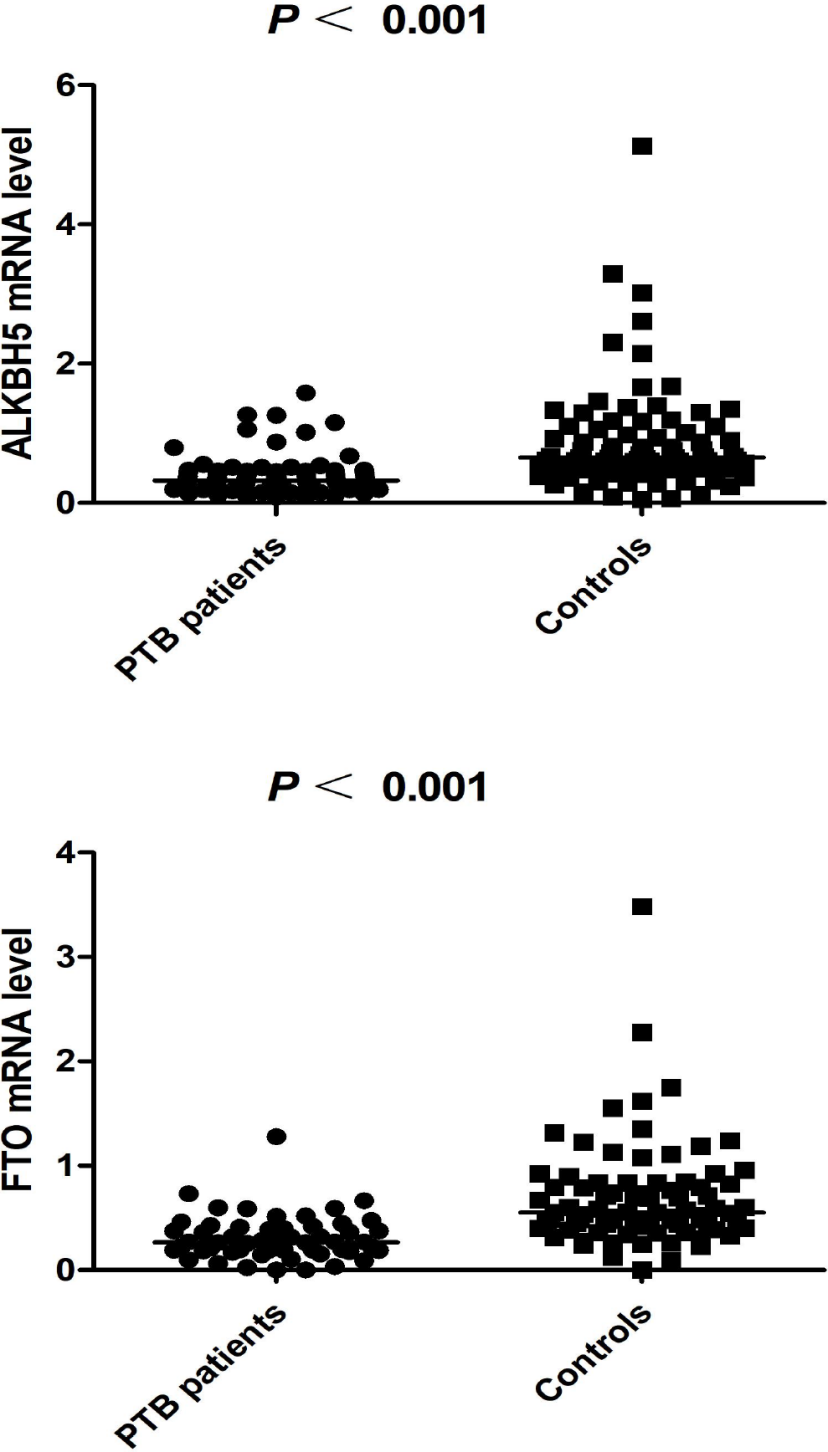
The expression levels of ALKBH5, FTO in PTB patients and controls (The subjects were selected from 449 patients and 463 controls).

The ALKBH5 expression level was detected in 77 PTB patients and 83 healthy controls, and the FTO expression level was detected in 57 PTB patients and 83 healthy controls. The data was summarized in **Figure 1**, and we found that ALKBH5, FTO expression levels were significantly decreased in PTB patients in comparison to healthy controls (all *P* <0.001). This study also explored the relationship between ALKBH5, FTO levels and some clinical features in PTB patients. The results demonstrated that ALKBH5 level was significantly increased in PTB patients with drug resistance when compared the PTB patients without drug resistance (*P*=0.022) (**Table 3**). Moreover, FTO level was significantly decreased in PTB patients with sputum smear-positive than that in the PTB patients with sputum smear-negative (*P*=0.036), and FTO level was negatively correlated with ESR (*P* = 0.038) (**Table 4**).

**Table 3:** The association between ALKBH5, FTO levels and clinical manifestations in PTB patients.

Group	+/-	ALKBH5			FTO		
		N	ALKBH5 level	*P* value	N	FTO level	*P* value
fever	+	15	0.262 (0.199,0.454)	0.375	12	0.232 (0.115,0.388)	0.457
	–	62	0.330 (0.231,0.463		45	0.266 (0.193,0.410)	
drug resistance	+	5	1.017 (0.338,1.323)	**0.022**	3	0.364 (0.192,0.589)	0.453
	–	72	0.310 (0.204,0.452)		54	0.263 (0.187,0.402)	
DILI	+	8	0.283 (0.209,0.414)	0.548	6	0.236 (0.166,0.367)	0.550
	–	69	0.339 (0.213,0.460)		51	0.266 (0.188,0.420)	
pulmonary infection	+	11	0.202 (0.177,0.291)	0.023	8	0.204 (0.092,0.294)	0.066
	–	66	0.355 (0.236,0.463)		49	0.270 (0.191,0.424)	
hypoproteinemia	+	16	0.220 (0.197,0.332)	0.022	11	0.215 (0.088,0.364)	0.093
	–	61	0.359 (0.247,0.473)		46	0.371 (0.192,0.422)	
leukopenia	+	6	0.344 (0.201,0.707)	0.718	6	0.250 (0.159,0.463)	0.897
	–	71	0.311 (0.220,0.457)		51	0.266 (0.190,0.410)	
sputum smear	+	26	0.253 (0.188,0.411)	0.058	17	0.193 (0.102,0.318)	**0.036**
	–	51	0.350 (0.251,0.469)		40	0.272 (0.194,0.440)	

+/-: with/without; median (interquartile range); Bold value means P < 0.05.

**Table 4:** The correlation between ALKBH5, FTO levels and clinical parameters in PTB patients.

Clinical parameters	ALKBH5 level			FTO level	
	*r_s_*	*P* value		*r_s_*	*P* value
ESR	-0.049	0.676		-0.278	0.038
TBIL	-0.170	0.145		-0.093	0.497
ALT	0.059	0.610		0.041	0.764
AST	0.063	0.585		0.045	0.741

r_s:_Spearman’s rank correlation coefficient.

### Association of ALKBH5, FTO levels with their different genotypes in PTB patients

We also analyzed whether *ALKBH5*, *FTO* genes variations affected their levels, respectively. As shown in **Table 5**, there might be some differences in ALKBH5, FTO expression levels among different genotypes, while no difference was reached statistical significance (all *P* > 0.05).

**Table 5:** Association between *ALKBH5*, *FTO* genes SNPs and their expression levels.

ALKBH5 SNP	Genotype	number	ALKBH5 level	P value
rs8400	AA	6	0.344 (0.152,0.566)	0.907
	GA	27	0.275 (0.206,0.445)	
	GG	23	0.311 (0.195,0.417)	
rs9913266	AA	5	0.311 (0.148,0.474)	0.987
	AG	26	0.286 (0.201,0.461)	
	GG	25	0.291 (0.199,0.427)	
rs12936694	AA	50	0.290 (0.196,0.441)	0.412
	AG	6	0.317 (0.247,0.599)	
	GG	0	–	
rs4925144	CC	29	0.311 (0.201,0.431)	0.845
	CT	21	0.262 (0.187,0.456)	
	TT	6	0.344 (0.201,0.566)	
FTO SNP	Genotype	number	FTO level	P value
rs6499640	GG	30	0.251 (0.181,0.402)	0.746
	GA	11	0.266 (0.188,0.389)	
	AA	0	–	
rs8047395	AA	14	0.252 (0.197,0.423)	0.674
	GA	20	0.237 (0.147,0.383)	
	GG	7	0.282 (0.192,0.376)	
rs1121980	GG	30	0.241 (0.177,0.365)	0.167
	GA	11	0.364 (0.193,0.460)	
	AA	0	–	
rs9939609	TT	34	0.241 (0.177,0.367)	0.127
	TA	7	0.389 (0.215,0.460)	
	AA	0	–	

Median (interquartile range).

## Discussion

Except for environmental factors, socioeconomic factors, host gene variation was also an important risk factor for PTB development. In recent years, many studies had proposed the potential contribution roles of m6A-related genes SNPs in the risk of multiple diseases, especially cancer (Lin et al., 2020; Ruan et al., 2021; Chen et al., 2021). Herein, we selected two main m6A demethylases (ALKBH5, FTO), and attempted to investigate whether multiple SNPs in *ALKBH5*, *FTO* genes were linked to the risk of PTB. According to our results, no SNP was found to be associated with the susceptibility to PTB, while *ALKBH5*, *FTO* genes variations might play certain influence on some clinical manifestations of PTB patients. It should be noted that this was the first study for revealing the abnormally reduced levels of ALKBH5, FTO in patients with PTB.

At present, the studies regarding ALKBH5, FTO genes were mainly focused on cancer, and ALKBH5, FTO had been identified as important malignant regulators of tumor cell phenotypes and therapeutic responses (Lan et al., 2020; Zhang et al., 2021). Some previous studies speculated that SNPs located at the key regulatory sites of *ALKBH5*, *FTO* might affect the development of disease by influencing host gene expression (Ren et al., 2021; Li et al., 2022). On the other hand, *ALKBH5*, *FTO* genes exerted an important role in the immune response to MTB infection, their gene variations might also affect PTB risk. The *FTO* gene rs9939609 polymorphisms was significantly associated with obesity in different populations, including Chinese population (Tan et al., 2008). Similar to other malnutrition states, obesity was also known to impair immune function, affect leukocyte counts and alter cell-mediated immune responses (de Heredia et al., 2012). Moreover, the impaired immune function could result in to an increased susceptibility of the host to pathogens such as MTB, coxsackie virus (Karlsson and Beck, 2010). Hence, it had been speculated that the *FTO* gene polymorphism might play important roles in the risk of immune-related human infectious diseases such as PTB, and the roles on PTB occurs through obesity-related immunocompetence. Given that the roles in *FTO*-SNPs in immune-related infectious, we have reason to believe that *ALKBH5* gene SNPs exert a similar role. Feng et al. found that compared with the common genotype TT, FTO rs9939609 AA genotype was strongly associated with a significantly increased risk of PTB in Chinese population (Feng et al., 2014). However, rs9939609 variant showed no significant difference in genotype, allele frequencies between PTB patients and controls in Iranian population in another study (Naderi et al., 2017). These two studies differ in the number of participants, race, experimental methods, which might lead to inconsistent results. In this study, we similarly did not find any significant association between rs9939609 variant and PTB susceptibility, and the same results were also observed for *FTO* rs6499640, rs8047395, rs1121980. Compared with single SNP, haplotypes tend to had a stronger power of predicting disease-related genes (Chen et al., 2018), and we detected a significantly lower frequency of *FTO* GAAA haplotype in PTB patients. This partially supported the hypothesis that *FTO* genetic polymorphisms might affect the host’s susceptibility to PTB, while the specific mechanisms need to be further explored by well-designed studies. Our study was the first to assessed the association of *ALKBH5* gene SNPs (rs8400, rs9913266, rs12936694, rs4925144) with PTB susceptibility, however no significant association was found. This provided some clues for future study on the role of *ALKBH5* gene variation in PTB development.

In addition to disease susceptibility, several host gene variations had been shown to affect the occurrence of the clinical manifestations of PTB patients. Li et al. revealed that *lncRNA NEAT1* rs3825071 variant was significantly related to the sputum smear-positive among PTB patients (Li et al., 2022). Similarly, our results provided strong evidence of significant association between rs8400 AA genotype, A allele, rs9913266 A allele frequencies and sputum smear-positive. This suggested that these SNP might contribute to the hierarchical management of PTB patients to develop different and appropriate therapeutic schedule. In addition, the results of another study suggested that *CYP27A1* rs17470271, rs933994 variants might affect the development of leukopenia, drug resistance in PTB patients, respectively (Zhang et al., 2021). In the present study, we found that rs8400, rs4925144 variants respectively affected the risk of hypoproteinemia, leukopenia. Furthermore, *ALKBH5* rs9913266 A allele was closely related to the occurrence of DILI, and *FTO* rs8047395 AA genotype, A allele frequencies were significantly decreased in PTB patients with drug resistance. Based on these results, it was reasonable to speculate that *ALKBH5*, *FTO* genes variations were closely related to the development process of PTB, and these SNPs could be used as potential indicators for some specific clinical manifestations of PTB patients.

It was of a great necessity to investigate the m6A demethylase expression level, and some studies found that the abnormally expressed level of ALKBH5, FTO were related to the pathogenesis of many diseases (Zhang et al., 2017; Guimarães-Teixeira et al., 2021; Deng et al., 2022). The study by Deng et al. showed that ALKBH5 level was downregulated in both PBMCs and T cells in systemic lupus erythematosus patients, and the overexpression of ALKBH5 could promoted apoptosis and inhibited the proliferation of T cells (Deng et al., 2022). The decreased *FTO* mRNA levels had been proved to be associated with a poor prognosis of renal cell carcinoma (36). Hence, this case-control study was conducted to detect the ALKBH5, FTO mRNA levels in PBMC from PTB patients, and revealed that the ALKBH5, FTO expression levels were significantly decreased in PTB patients in comparison with controls. This provided key evidence to confirm that ALKBH5, FTO were involved in the occurrence of PTB, and their levels could be used as a potential, auxiliary indicator for PTB diagnosis, which should be explored and verified by further studies. Our study also showed that ALKBH5 level was significantly associated with drug resistance, and FTO level was significantly associated with sputum smear-positive, ESR in PTB patients. These significant associations between ALKBH5, FTO levels and the clinical manifestations were contributed to understand the role of ALKBH5, FTO in the development of PTB.

The strengths of this study included the good design and novel topic. We simultaneously examined *ALKBH5, FTO* gene variations, expression levels, and analyzed their association with PTB patients. However, some limitations should not be neglected. First, the sample size could be increased to get more reliable results, although our study appeared to be adequate, and the statistical power of every SNP exceeded 85%. Second, we could not analyze the interaction between genetic variations and environmental factors due to no environment information. Third, other SNPs with important functions might not be selected. The studies with multiple centers, large samples were needed to accurately assess the mechanism of ALKBH5, FTO genes in the pathogenesis of PTB in the future.

## Conclusion

In summary, a significantly decreased frequency of GAAA haplotype in PTB demonstrated that *FTO* gene polymorphisms might associated with PTB susceptibility, whereas *ALKBH5* SNPs had no predictive value in evaluating PTB susceptibility. Several SNPs in *ALKBH5, FTO* genes were associated with the clinical features of PTB patients such as drug resistance, DILI, hypoproteinemia, and *ALKBH5, FTO* levels were significantly lower in PTB patients. This study will help to further understand the pathogenesis of PTB, which could contribute to formulated more appropriate clinical treatment measures.

## Data availability statement

The original contributions presented in the study are included in the article/**Supplementary Materials**. Further inquiries can be directed to the corresponding author.

## Ethics statement

This study was approved by the Ethics Committee of Anhui Medical University (20200250). The patients/participants provided their written informed consent to participate in this study.

## Author contributions

T-PZ and H-ML designed the study. H-ML conducted the experiment. RL performed the statistical analyses. L-JW participated in sample collection. T-PZ and H-ML drafted the manuscript. All authors contributed to the article and approved the submitted version.
