# Supplementary material for: Impact of m6A demethylase (ALKBH5, FTO) genetic polymorphism and expression levels on the development of pulmonary tuberculosis
Source: Front Cell Infect Microbiol. 2022 Dec 22;12:1074380. doi: 10.3389/fcimb.2022.1074380 (PMC9817133; doi:10.3389/fcimb.2022.1074380)
Supplement: Supplementary file 1 [file Table_1.doc]

**Table S1** Association between *ALKBH5*, *FTO* genes polymorphisms and PTB susceptibility

| SNP | Analyze model | | PTB patients | Controls | *P* value | OR (95% CI) |
| --- | --- | --- | --- | --- | --- | --- |
| *ALKBH5* | | | | | | |
| rs8400 | Genotypes | AA | 74(16.48) | 74(15.98) | 0.891 | 1.028(0.692,1.527) |
|  |  | GA | 232(51.67) | 242(52.27) | 0.922 | 0.985(0.736,1.320) |
|  |  | GG | 143(31.85) | 147(31.75) | Reference | |
|  | Alleles | A | 380(42.32) | 390(42.12) | 0.931 | 1.005(0.902,1.119) |
|  |  | G | 518(57.68) | 536(57.88) | Reference | |
|  | Dominant model | GG | 143(31.85) | 147(31.75) | 0.974 | 0.999(0.914,1.091) |
|  |  | GA+AA | 306(68.15) | 316(68.25) | Reference | |
|  | Recessive model | AA | 74(16.48) | 74(15.98) | 0.838 | 1.031(0.768,1.385) |
|  |  | GA+GG | 375(83.52) | 389(84.02) | Reference | |
| rs9913266 | Genotypes | AA | 41(9.13) | 41(8.86) | 0.927 | 0.978(0.611,1.566) |
|  |  | AG | 179(39.87) | 198(42.76) | 0.378 | 0.884(0.673,1.162) |
|  |  | GG | 229(51.00) | 224(48.38) | Reference | |
|  | Alleles | A | 261(29.06) | 280(30.24) | 0.583 | 1.017(0.958,1.079) |
|  |  | G | 637(70.94) | 646(69.76) | Reference | |
|  | Dominant model | GG | 229(51.00) | 224(48.38) | 0.428 | 1.054(0.925,1.201) |
|  |  | AG+AA | 220(49.00) | 239(51.62) | Reference | |
|  | Recessive model | AA | 41(9.13) | 41(8.86) | 0.884 | 0.997(0.957,1.039) |
|  |  | AG+GG | 408(90.87) | 422(91.14) | Reference | |
| rs12936694 | Genotypes | GG | 2(0.45) | 1(0.22) | 0.574 | 1.995(0.180,22.091) |
|  |  | AG | 62(13.81) | 78(16.85) | 0.209 | 0.793(0.552,1.139) |
|  |  | AA | 385(85.75) | 384(82.94) | Reference | |
|  | Alleles | G | 66(7.35) | 80(8.64) | 0.310 | 1.014(0.987,1.042) |
|  |  | A | 832(92.65) | 846(91.36) | Reference | |
|  | Dominant model | AA | 385(85.75) | 384(82.94) | 0.244 | 0.835(0.617,1.131) |
|  |  | AG+GG | 64(14.25) | 79(17.06) | Reference | |
|  | Recessive model | GG | 2(0.45) | 1(0.22) | 0.545 | 2.062(0.188,22.664) |
|  |  | AG+AA | 447(99.55) | 462(99.78) | Reference | |
| rs4925144 | Genotypes | TT | 55(12.25) | 52(11.23) | 0.561 | 1.135(0.740,1.740) |
|  |  | CT | 203(45.21) | 206(44.49) | 0.691 | 1.058(0.802,1.394) |
|  |  | CC | 191(42.54) | 205(44.28) | Reference | |
|  | Alleles | T | 313(34.86) | 310(33.48) | 0.535 | 1.041(0.917,1.183) |
|  |  | C | 585(65.14) | 616(66.52) | Reference | |
|  | Dominant model | CC | 191(42.54) | 205(44.28) | 0.597 | 0.961(0.828,1.114) |
|  |  | CT+TT | 258(57.46) | 258(55.72) | Reference | |
|  | Recessive model | TT | 55(12.25) | 52(11.23) | 0.633 | 1.091(0.764,1.557) |
|  |  | CT+CC | 394(87.75) | 411(88.77) | Reference | |
| *FTO* | | | | | | |
| rs6499640 | Genotypes | AA | 5(1.11) | 12(2.59) | 0.114 | 0.427(0.149,1.227) |
|  |  | GA | 129(28.73) | 128(27.65) | 0.824 | 1.033(0.774,1.381) |
|  |  | GG | 315(70.16) | 323(69.76) | Reference | |
|  | Alleles | A | 139(15.48) | 152(16.41) | 0.585 | 0.943(0.764,1.164) |
|  |  | G | 759(84.52) | 774(83.59) | Reference | |
|  | Dominant model | GG | 315(70.16) | 323(69.76) | 0.897 | 1.006(0.924,1.095) |
|  |  | GA+AA | 134(29.84) | 140(30.24) | Reference | |
|  | Recessive model | AA | 5(1.11) | 12(2.59) | 0.099 | 0.430(0.148,1.211) |
|  |  | GA+GG | 444(98.89) | 451(97.41) | Reference | |
| rs8047395 | Genotypes | GG | 68(15.14) | 68(14.69) | 0.386 | 1.192(0.801,1.775) |
|  |  | GA | 225(50.11) | 209(45.14) | 0.085 | 1.284(0.966,1.705) |
|  |  | AA | 156(34.74) | 186(40.17) | Reference | |
|  | Alleles | G | 361(40.20) | 345(37.26) | 0.197 | 1.097(0.961,1.211) |
|  |  | A | 537(59.8) | 581(62.74) | Reference | |
|  | Dominant model | AA | 156(34.74) | 186(40.17) | 0.090 | 0.865(0.731,1.024) |
|  |  | GA+GG | 293(65.26) | 277(59.83) | Reference | |
|  | Recessive model | GG | 68(15.14) | 68(14.69) | 0.846 | 1.031(0.720,1.493) |
|  |  | GA+AA | 381(84.86) | 395(85.31) | Reference | |
| rs1121980 | Genotypes | AA | 13(2.90) | 16(3.46) | 0.602 | 0.820(0.388,1.731) |
|  |  | GA | 102(22.72) | 110(23.76) | 0.673 | 0.936(0.687,1.274) |
|  |  | GG | 334(74.39) | 337(72.79) | Reference | |
|  | Alleles | A | 128(14.25) | 142(15.33) | 0.516 | 0.930(0.746,1.159) |
|  |  | G | 770(85.75) | 784(84.67) | Reference | |
|  | Dominant model | GG | 334(74.39) | 337(72.79) | 0.583 | 1.022(0.946,1.105) |
|  |  | GA+AA | 115(25.61) | 126(27.21) | Reference | |
|  | Recessive model | AA | 13(2.90) | 16(3.46) | 0.630 | 0.838(0.396,1.752) |
|  |  | GA+GG | 436(97.10 | 447(96.54) | Reference | |
| rs9939609 | Genotypes | AA | 4(0.89) | 5(1.08) | 0.732 | 0.794(0.211,2.978) |
|  |  | TA | 73(16.26) | 89(19.22) | 0.236 | 0.814(0.578,1.145) |
|  |  | TT | 372(82.85) | 369(79.7) | Reference | |
|  | Alleles | A | 81(9.02) | 99(10.69) | 0.232 | 0.844(0.638,1.115) |
|  |  | T | 817(90.98) | 827(89.31) | Reference | |
|  | Dominant model | TT | 372(82.85) | 369(79.70 | 0.223 | 1.040(0.977,1.106) |
|  |  | TA+AA | 77(17.15) | 94(20.30 | Reference | |
|  | Recessive model | AA | 4(0.89) | 5(1.08) | 0.773 | 0.825(0.223,3.052) |
|  |  | TA+TT | 445(99.11) | 458(98.92) | Reference | |

**Table S2** Association between *ALKBH5*, *FTO* gene polymorphism and some clinical manifestations in PTB patients

| SNP | Allele | Clinical features | Group | Genotype n (%) | | | *P* value | Allele n (%) | | *P* value |
| --- | --- | --- | --- | --- | --- | --- | --- | --- | --- | --- |
|  | (M/m) |  |  | MM | Mm | mm |  | M | m |  |
| *ALKBH5* | | | | | | | | | | |
| rs8400 | G/A | fever | + | 24(34.78) | 34(49.28) | 11(15.94) | 0.850 | 83(59.71) | 56(40.29) | 0.607 |
|  |  |  | - | 119(31.32) | 198(52.11) | 63(16.58) |  | 436(57.37) | 324(42.63) |  |
|  |  | drug resistance | + | 25(33.78) | 36(48.65) | 13(17.57) | 0.850 | 86(58.11) | 62(41.89) | 0.909 |
|  |  |  | - | 118(31.47) | 196(52.27) | 61(16.27) |  | 432(57.6) | 318(42.4) |  |
|  |  | DILI | + | 28(42.42) | 30(45.45) | 8(12.12) | 0.123 | 86(65.15) | 46(34.85) | 0.060 |
|  |  |  | - | 115(30.03) | 202(52.74) | 66(17.23) |  | 432(56.4) | 334(43.6) |  |
|  |  | Pulmonary infection | + | 25(30.86) | 39(48.15) | 17(20.99) | 0.476 | 89(54.94) | 73(45.06) | 0.435 |
|  |  |  | - | 118(32.07) | 193(52.45) | 57(15.49) |  | 429(58.29) | 307(41.71) |  |
|  |  | hypoproteinemia | + | 12(31.58) | 14(36.84) | 12(31.58) | **0.023** | 38(50.00) | 38(50.00) | 0.156 |
|  |  |  | - | 131(31.87) | 218(53.04) | 62(15.09) |  | 480(58.39) | 342(41.61) |  |
|  |  | leukopenia | + | 6(20.00) | 18(60.00) | 6(20.00) | 0.352 | 30(50.00) | 30(50.00) | 0.212 |
|  |  |  | - | 137(32.7) | 214(51.07) | 68(16.23) |  | 488(58.23) | 350(41.77) |  |
|  |  | sputum smear | + | 99(34.62) | 152(53.15) | 35(12.24) | **0.015** | 350(61.19) | 222(38.81) | **0.019** |
|  |  |  | - | 35(28.23) | 60(48.39) | 29(23.39) |  | 130(52.42) | 118(47.58) |  |
| rs9913266 | G/A | fever | + | 37(53.62) | 22(31.88) | 10(14.49) | 0.137 | 96(69.57) | 42(30.43) | 0.700 |
|  |  |  | - | 192(50.53) | 157(41.32) | 31(8.16) |  | 541(71.18) | 219(28.82) |  |
|  |  | drug resistance | + | 41(55.41) | 27(36.49) | 6(8.11) | 0.707 | 109(73.65) | 39(26.35) | 0.426 |
|  |  |  | - | 188(50.13) | 152(40.53) | 35(9.33) |  | 528(70.40) | 222(29.60) |  |
|  |  | DILI | + | 26(39.39) | 32(48.48) | 8(12.12) | 0.120 | 84(63.64) | 48(36.36) | 0.046 |
|  |  |  | - | 203(53.00) | 147(38.38) | 33(8.62) |  | 553(72.19) | 213(27.81) |  |
|  |  | pulmonary infection | + | 185(50.27) | 148(40.22) | 35(9.51) | 0.742 | 518(70.38) | 218(29.62) | 0.435 |
|  |  |  | - | 44(54.32) | 31(38.27) | 6(7.41) |  | 119(73.46) | 43(26.54) |  |
|  |  | hypoproteinemia | + | 23(60.53) | 11(28.95) | 4(10.53) | 0.356 | 57(75.00) | 19(25.00) | 0.415 |
|  |  |  | - | 206(50.12) | 168(40.88) | 37(9.00) |  | 580(70.56) | 242(29.44) |  |
|  |  | leukopenia | + | 18(60.00) | 10(33.33) | 2(6.67) | 0.586 | 46(76.67) | 14(23.33) | 0.311 |
|  |  |  | - | 211(50.36) | 169(40.33) | 39(9.31) |  | 591(70.53) | 247(29.47) |  |
|  |  | sputum smear | + | 71(57.26) | 44(35.48) | 9(7.26) | 0.141 | 186(75.00) | 62(25.00) | 0.049 |
|  |  |  | - | 134(46.85) | 122(42.66) | 30(10.49) |  | 390(68.18) | 182(31.82) |  |
| rs12936694 | A/G | fever | + | 60(86.96) | 8(11.59) | 1(1.45) | 0.342 | 128(92.75) | 10(7.25) | 0.960 |
|  |  |  | - | 325(85.53) | 54(14.21) | 1(0.26) |  | 704(92.63) | 56(7.37) |  |
|  |  | drug resistance | + | 65(87.84) | 9(12.16) | 0(0) | 0.736 | 139(93.92) | 9(6.08) | 0.518 |
|  |  |  | - | 320(85.33) | 53(14.13) | 2(0.53) |  | 693(92.40) | 57(7.60) |  |
|  |  | DILI | + | 58(87.88) | 8(12.12) | 0(0) | 0.761 | 124(93.94) | 8(6.06) | 0.539 |
|  |  |  | - | 327(85.38) | 54(14.10) | 2(0.52) |  | 708(92.43) | 58(7.57) |  |
|  |  | pulmonary infection | + | 70(86.42) | 10(12.35) | 1(1.23) | 0.463 | 150(92.59) | 12(7.41) | 0.975 |
|  |  |  | - | 315(85.6) | 52(14.13) | 1(0.27) |  | 682(92.66) | 54(7.34) |  |
|  |  | hypoproteinemia | + | 35(92.11) | 3(7.89) | 0(0) | 0.489 | 73(96.05) | 3(3.95) | 0.235 |
|  |  |  | - | 350(85.16) | 59(14.36) | 2(0.49) |  | 759(92.34) | 63(7.66) |  |
|  |  | leukopenia | + | 25(83.33) | 5(16.67) | 0(0) | 0.837 | 55(91.67) | 5(8.33) | 0.762 |
|  |  |  | - | 360(85.92) | 57(13.60) | 2(0.48) |  | 777(92.72) | 61(7.28) |  |
|  |  | sputum smear | + | 104(83.87) | 20(16.13) | 0(0) | 0.509 | 228(91.94) | 20(8.06) | 0.526 |
|  |  |  | - | 247(86.36) | 39(13.64) | 0(0) |  | 533(93.18) | 39(6.82) |  |
| rs4925144 | C/T | fever | + | 34(49.28) | 24(34.78) | 11(15.94) | 0.154 | 92(66.67) | 46(33.33) | 0.683 |
|  |  |  | - | 157(41.32) | 179(47.11) | 44(11.58) |  | 493(64.87) | 267(35.13) |  |
|  |  | drug resistance | + | 33(44.59) | 30(40.54) | 11(14.86) | 0.603 | 96(64.86) | 52(35.14) | 0.938 |
|  |  |  | - | 158(42.13) | 173(46.13) | 44(11.73) |  | 489(65.20) | 261(34.80) |  |
|  |  | DILI | + | 33(50) | 28(42.42) | 5(7.58) | 0.283 | 94(71.21) | 38(28.79) | 0.113 |
|  |  |  | - | 158(41.25) | 175(45.69) | 50(13.05) |  | 491(64.1) | 275(35.9) |  |
|  |  | pulmonary infection | + | 33(40.74) | 36(44.44) | 12(14.81) | 0.734 | 102(62.96) | 60(37.04) | 0.520 |
|  |  |  | - | 158(42.93) | 167(45.38) | 43(11.68) |  | 483(65.63) | 253(34.38) |  |
|  |  | hypoproteinemia | + | 14(36.84) | 15(39.47) | 9(23.68) | 0.080 | 43(56.58) | 33(43.42) | 0.101 |
|  |  |  | - | 177(43.07) | 188(45.74) | 46(11.19) |  | 542(65.94) | 280(34.06) |  |
|  |  | leukopenia | + | 7(23.33) | 21(70.00) | 2(6.67) | 0.019 | 35(58.33) | 25(41.67) | 0.252 |
|  |  |  | - | 184(43.91) | 182(43.44) | 53(12.65) |  | 550(65.63) | 288(34.37) |  |
|  |  | sputum smear | + | 47(37.90) | 58(46.77) | 19(15.32) | 0.160 | 152(61.29) | 96(38.71) | 0.062 |
|  |  |  | - | 131(45.80) | 127(44.41) | 28(9.79) |  | 389(68.01) | 183(31.99) |  |
| *FTO* | | | | | | | | | | |
| rs6499640 | G/A | fever | + | 50(72.46) | 18(26.09) | 1(1.45) | 0.843 | 118(85.51) | 20(14.49) | 0.728 |
|  |  |  | - | 265(69.74) | 111(29.21) | 4(1.05) |  | 641(84.34) | 119(15.66) |  |
|  |  | drug resistance | + | 57(77.03) | 17(22.97) | 0(0) | 0.272 | 131(88.51) | 17(11.49) | 0.142 |
|  |  |  | - | 258(68.80) | 112(29.87) | 5(1.33) |  | 628(83.73) | 122(16.27) |  |
|  |  | DILI | + | 48(72.73) | 17(25.76) | 1(1.52) | 0.809 | 113(85.61) | 19(14.39) | 0.709 |
|  |  |  | - | 267(69.71) | 112(29.24) | 4(1.04) |  | 646(84.33) | 120(15.67) |  |
|  |  | pulmonary infection | + | 56(69.14) | 24(29.63) | 1(1.23) | 0.973 | 136(83.95) | 26(16.05) | 0.824 |
|  |  |  | - | 259(70.38) | 105(28.53) | 4(1.09) |  | 623(84.65) | 113(15.35) |  |
|  |  | hypoproteinemia | + | 30(78.95) | 8(21.05) | 0(0) | 0.412 | 68(89.47) | 8(10.53) | 0.212 |
|  |  |  | - | 285(69.34) | 121(29.44) | 5(1.22) |  | 691(84.06) | 131(15.94) |  |
|  |  | leukopenia | + | 22(73.33) | 7(23.33) | 1(3.33) | 0.408 | 51(85.00) | 9(15.00) | 0.915 |
|  |  |  | - | 293(69.93) | 122(29.12) | 4(0.95) |  | 708(84.49) | 130(15.51) |  |
|  |  | sputum smear | + | 94(75.81) | 29(23.39) | 1(0.81) | 0.165 | 217(87.50) | 31(12.50) | 0.074 |
|  |  |  | - | 190(66.43) | 92(32.17) | 4(1.4) |  | 472(82.52) | 100(17.48) |  |
| rs8047395 | A/G | fever | + | 26(37.68) | 31(44.93) | 12(17.39) | 0.634 | 83(60.14) | 55(39.86) | 0.928 |
|  |  |  | - | 130(34.21) | 194(51.05) | 56(14.74) |  | 454(59.74) | 306(40.26) |  |
|  |  | drug resistance | + | 16(21.62) | 42(56.76) | 16(21.62) | **0.022** | 74(50.00) | 74(50.00) | **0.008** |
|  |  |  | - | 140(37.33) | 183(48.8) | 52(13.87) |  | 463(61.73) | 287(38.27) |  |
|  |  | DILI | + | 16(24.24) | 38(57.58) | 12(18.18) | 0.151 | 70(53.03) | 62(46.97) | 0.086 |
|  |  |  | - | 140(36.55) | 187(48.83) | 56(14.62) |  | 467(60.97) | 299(39.03) |  |
|  |  | pulmonary infection | + | 23(28.40) | 47(58.02) | 11(13.58) | 0.281 | 93(57.41) | 69(42.59) | 0.493 |
|  |  |  | - | 133(36.14) | 178(48.37) | 57(15.49) |  | 444(60.33) | 292(39.67) |  |
|  |  | hypoproteinemia | + | 15(39.47) | 17(44.74) | 6(15.79) | 0.772 | 47(61.84) | 29(38.16) | 0.704 |
|  |  |  | - | 141(34.31) | 208(50.61) | 62(15.09) |  | 490(59.61) | 332(40.39) |  |
|  |  | leukopenia | + | 8(26.67) | 17(56.67) | 5(16.67) | 0.629 | 33(55.00) | 27(45.00) | 0.432 |
|  |  |  | - | 148(35.32) | 208(49.64) | 63(15.04) |  | 504(60.14) | 334(39.86) |  |
|  |  | sputum smear | + | 45(36.29) | 59(47.58) | 20(16.13) | 0.740 | 149(60.08) | 99(39.92) | 0.864 |
|  |  |  | - | 96(33.57) | 148(51.75) | 42(14.69) |  | 340(59.44) | 232(40.56) |  |
| rs1121980 | G/A | fever | + | 49(71.01) | 19(27.54) | 1(1.45) | 0.461 | 117(84.78) | 21(15.22) | 0.725 |
|  |  |  | - | 285(75.00) | 83(21.84) | 12(3.16) |  | 653(85.92) | 107(14.08) |  |
|  |  | drug resistance | + | 60(81.08) | 13(17.57) | 1(1.35) | 0.317 | 133(89.86) | 15(10.14) | 0.117 |
|  |  |  | - | 274(73.07) | 89(23.73) | 12(3.20) |  | 637(84.93) | 113(15.07) |  |
|  |  | DILI | + | 53(80.30) | 12(18.18) | 1(1.52) | 0.455 | 118(89.39) | 14(10.61) | 0.194 |
|  |  |  | - | 281(73.37) | 90(23.50) | 12(3.13) |  | 652(85.12) | 114(14.88) |  |
|  |  | pulmonary infection | + | 58(71.6) | 21(25.93) | 2(2.47) | 0.736 | 137(84.57) | 25(15.43) | 0.636 |
|  |  |  | - | 276(75.00) | 81(22.01) | 11(2.99) |  | 633(86.01) | 103(13.99) |  |
|  |  | hypoproteinemia | + | 28(73.68) | 10(26.32) | 0(0) | 0.486 | 66(86.84) | 10(13.16) | 0.775 |
|  |  |  | - | 306(74.45) | 92(22.38) | 13(3.16) |  | 704(85.64) | 118(14.36) |  |
|  |  | leukopenia | + | 23(76.67) | 6(20.00) | 1(3.33) | 0.929 | 52(86.67) | 8(13.33) | 0.833 |
|  |  |  | - | 311(74.22) | 96(22.91) | 12(2.86) |  | 718(85.68) | 120(14.32) |  |
|  |  | sputum smear | + | 91(73.39) | 33(26.61) | 0(0) | 0.089 | 215(86.69) | 33(13.31) | 0.795 |
|  |  |  | - | 215(75.17) | 62(21.68) | 9(3.15) |  | 492(86.01) | 80(13.99) |  |
| rs9939609 | T/A | fever | + | 53(76.81) | 15(21.74) | 1(1.45) | 0.341 | 121(87.68) | 17(12.32) | 0.141 |
|  |  |  | - | 319(83.95) | 58(15.26) | 3(0.79) |  | 696(91.58) | 64(8.42) |  |
|  |  | drug resistance | + | 66(89.19) | 8(10.81) | 0(0) | 0.242 | 140(94.59) | 8(5.41) | 0.093 |
|  |  |  | - | 306(81.6) | 65(17.33) | 4(1.07) |  | 677(90.27) | 73(9.73) |  |
|  |  | DILI | + | 59(89.39) | 7(10.61) | 0(0) | 0.271 | 125(94.7) | 7(5.30) | 0.107 |
|  |  |  | - | 313(81.72) | 66(17.23) | 4(1.04) |  | 692(90.34) | 74(9.66) |  |
|  |  | pulmonary infection | + | 66(81.48) | 15(18.52) | 0(0) | 0.545 | 147(90.74) | 15(9.26) | 0.907 |
|  |  |  | - | 306(83.15) | 58(15.76) | 4(1.09) |  | 670(91.03) | 66(8.97) |  |
|  |  | hypoproteinemia | + | 32(84.21) | 6(15.79) | 0(0) | 0.825 | 70(92.11) | 6(7.89) | 0.720 |
|  |  |  | - | 340(82.73) | 67(16.3) | 4(0.97) |  | 747(90.88) | 75(9.12) |  |
|  |  | leukopenia | + | 25(83.33) | 5(16.67) | 0(0) | 0.865 | 55(91.67) | 5(8.33) | 0.848 |
|  |  |  | - | 347(82.82) | 68(16.23) | 4(0.95) |  | 762(90.93) | 76(9.07) |  |
|  |  | sputum smear | + | 105(84.68) | 19(15.32) | 0(0) | 0.616 | 229(92.34) | 19(7.66) | 0.555 |
|  |  |  | - | 237(82.87) | 47(16.43) | 2(0.70) |  | 521(91.08) | 51(8.92) |  |
